# Cetylpyridinium chloride promotes disaggregation of SARS-CoV-2 virus-like particles

**DOI:** 10.1080/20002297.2022.2030094

**Published:** 2022-01-24

**Authors:** Manuel Bañó-Polo, Luis Martínez-Gil, Manuel M. Sánchez del Pino, Alberto Massoli, Ismael Mingarro, Rubén Léon, Maria Jesus Garcia-Murria

**Affiliations:** a Department of Microbiology. Dentaid Research Center, Cerdanyola del Vallès, Barcelona, Spain; b Department of Biochemistry and Molecular Biology, Institute for Biotechnology and Biomedicine (BIOTECMED), University of Valencia, Valencia, Spain

**Keywords:** SARS-CoV-2, cetylpyridinium chloride, virology, virus-like particles, membrane disaggregation

## Abstract

**Background:**

SARS-CoV-2 is continuously disseminating worldwide. The development of strategies to break transmission is mandatory.

**Aim of the study:**

To investigate the potential of cetylpyridinium chloride (CPC) as a viral inhibitor.

**Methods:**

SARS-CoV-2 Virus Like-Particles (VLPs) were incubated with CPC, a potent surfactant routinely included in mouthwash preparations.

**Results:**

Concentrations of 0.05% CPC (w/v) commonly used in mouthwash preparations are sufficient to promote the rupture of SARS-CoV-2 VLP membranes.

**Conclusion:**

Including CPC in mouthwashes could be a prophylactic strategy to keep SARS-CoV-2 from spreading.

## Introduction

Coronavirus disease 2019 (COVID-19), an extraordinarily infectious human disease caused by SARS-CoV-2, has spread around the world at a speedy rate, causing a worldwide pandemic. SARS-CoV-2 is an enveloped virus with a positive-sense, single-stranded RNA genome (29,9 kb) [1]. SARS-CoV-2 genome encodes four major structural proteins: the spike (S) protein, the nucleocapsid (N) protein, the membrane (M) protein, and the envelope (E) protein, all of which are required to produce a structurally complete viral particle [2]. The structural N protein is responsible for coordinating the interaction between the viral RNA genome and the cytosolic C-terminal domain of the M protein in an RNA-independent manner [3]. The membrane-embedded M, E and S proteins play a key role in life cycle processes such as assembly, viral maturation and virus release and entry. Independently of the entry pathway, at some point during the SARS-CoV-2 life cycle, viruses must cross the cell membrane. For enveloped viruses, infection requires the fusion of the viral and cellular membranes. In the case of SARS-CoV-2, this membrane merge process is triggered by the interaction of the S protein with the human receptor ACE2 [4].

SARS-CoV-2 virus is detectable in saliva from infected individuals without symptoms or with mild symptoms [5], suggesting that a strategy of reducing the viral load in the oral cavity may reduce viral spread [6]. Several studies have shown the antiviral potential of mouthwashes, which decrease the infectivity of airborne viruses such as influenza and several coronaviruses, including SARS-CoV-2 [7]. In fact, the use of molecules such as cetylpyridinium chloride (CPC), commonly found in mouthwashes, has been postulated as a supplementary strategy to fight the transmission of viruses such as Influenza [8] or Herpesviruses [9], where the oral cavity plays an important role in spreading the virus. However, further clinical studies are needed to confirm this approach as a prophylactic strategy. Meanwhile, the underlying mechanism of the antiviral activity of the CPC molecule and mouth rinses containing this ingredient remains to be determined, although potential membrane degradation mechanisms by which mouth rinses inhibit the spread of SARS-CoV-2 have been postulated [10–12].

Here, using a mammalian expression system, we efficiently generated SARS-CoV-2 virus-like particles (VLPs). These particles contain all four structural proteins of the virus and have the size and morphology of SARS-CoV-2 virions [13]. Our results demonstrated that CPC significantly decreased the integrity of SARS-CoV-2 VLPs at a concentration as low as 0.05%.

## Materials and methods

### Plasmid constructs

Sequences encoding the S spike protein (Gen Bank: QHD43416.1), E protein (Gen Bank: QHD43418.1), M protein (Gen Bank: QHD43419.1), and N protein (Gen Bank: QHD43423.2) were synthesised by Invitrogen (GeneArt gene synthesis). Next, these DNA sequences were subcloned with a c-myc epitope at their N- or C-terminus into KpnI linearised pCAGGS plasmid using In-Fusion HD cloning kit (Takara) according to the manufacturer’s instructions. The sequence was verified by sequencing the plasmid DNA at Macrogen Company (Seoul, South Korea).

### Cells and culture conditions

Human embryonic kidney (HEK293T) cells (from American Type Cell Collection, Manassas, VA, USA) were maintained in Dulbecco’s modified Eagle’s medium (DMEM, Gibco) supplemented with 10% foetal bovine serum, 1% penicillin–streptomycin (Sigma Aldrich, St. Louis, MO, USA), and 1% MEM non-essential amino acids. The cells were maintained at 37°C and 5% CO_2_ conditions.

### Transient transfections

HEK293T cells were seeded in 100 mm dishes and grown in DMEM (Gibco) supplemented with 10% foetal bovine serum (FBS, Gibco) the day before transfection. After 24 h, DMEM medium was mixed with pCAGGS plasmids coding for SARS-CoV-2 structural S, M, E and N proteins (where N or M was c-myc tagged) (2 μg of each plasmid each) and transfected into HEK293T cells using Lipofectamine 2000 (Life Technologies) as previously described [14,15]. Transfection mixtures were added dropwise onto cells in DMEM + 3% FBS and incubated for 72 h.

### Purification of SARS-CoV-2 virus-like particles

Culture media from transfected cells containing VLPs was collected and centrifuged twice at 200 g for 10 min at room temperature. Supernatants were added to ultracentrifuge tubes with a 20% sucrose/Tris-buffer saline (TBS 20 mM Tris, pH approx. 7.4, 0.9% NaCl) cushion, followed by ultracentrifugation at 151.000 g for 2 h at 4°C in a SW41TI rotor (Beckman Coulter). Pellets, containing the VLPs, were resuspended in TBS buffer and ultracentrifuged at 112.000 g for 15 min at 4°C in a TLA55 rotor (Beckman Coulter) in the 20% sucrose/TBS cushion. Pellets containing the VLPs were collected.

To test the effect of CPC (Merck), purified VLPs were incubated with CPC (from 0.5% to 0.0005%) or Sodium dodecyl sulfate (SDS) (0.5%) for 1 min, both detergents dissolved in PBS (Both detergents were obtained as pure powder from Merck, USA). Samples were ultracentrifuged at 112.000 g for 15 min at 4°C in a TLA55 rotor (Beckman Coulter) in a 20% sucrose/TBS cushion. The supernatant and pellet were recovered and analysed by Western blot.

For electron microscopy visualisation, the VLPs collected from the medium were filtered using a 0.2 µm filter before the centrifugations and the TBS buffer was exchanged for TNE buffer (50 mM Tris-HCl, 100 mM NaCl, 0.5 mM EDTA, pH 7.4).

### Purification and analysis of proteins associated with SARS-CoV-2-VLPs

Supernatants from transfected cells containing VLPs were purified as described [16,17]. Doubly ultracentrifuged pellets were resuspended in PNGase-F Glycoprotein Denaturing Buffer (New England Biolabs), and the proteins within were deglycosylated following the manufacturer’s instructions. Removal of N-glycans is advisable because: 1. Glycosylation reduces trypsin efficiency. 2. Unmodified proteins have higher ionisation efficiency than their glycosylated counterparts. 3. Protein identification efficiency increases after deglycosylation. Next, samples were analysed by SDS-PAGE prior to their analysis by mass spectrometry (MS). The MS analysis was done at the Proteomics Core Facility of the University of Valencia. Four independent replicates were analysed. The HEK293T cell samples transfected with empty pCAGGS were used as a control.

### Sample preparation for mass spectrometry analysis

For the MS analysis, samples (four biological replicates) were run at 100 V for 30 min in a reducing discontinuous SDS-PAGE to concentrate the protein extract. Protein bands were excised from the gel, washed, reduced, S-alkylated with iodoacetamide, and in-gel digested with 500 ng of trypsin (overnight at 37°C) as described previously [18]. The digestion mixture was dried in a vacuum centrifuge and resuspended in 15 μL of 2% acetonitrile and 0.1% trifluoroacetic acid. As previously mentioned, the proteomic analysis was performed in the proteomics facility of SCSIE University of Valencia.

### Liquid chromatography and tandem mass spectrometry (LC–MS/MS)

An aliquot of each sample (3 µL) was loaded onto a trap column (ChromXP C18, 3 μm 120 Å, 350 μm, 0.5 mm; Eksigent) and desalted with 0.1% TFA at 5 µl/min during 5 min. The peptides were then loaded onto an analytical column (ACQUITY UPLC M-Class, HSS T3, 100 Å, 1.8 µm, 75 µm × 250 mm; Waters) equilibrated in 5% acetonitrile 0.1% FA (formic acid). Elution was carried out with a linear gradient of 7 a 40% B in A for 20 min. (A: 0.1% FA; B: ACN, 0.1% FA) at a flow rate of 300 nL/min. Peptides were analysed in a mass spectrometer microESI qQTOF (6600plus TripleTOF, ABSCIEX). The sample was ionised in a Source Type: Optiflow <1 uL Nano applying 3.0 kV to the spray emitter at 200°C. Analysis was carried out in a data-dependent mode. Survey MS1 scans were acquired from 350 to 1400 m/z for 250 ms. The quadrupole resolution was set to ‘LOW’ for MS2 experiments, which were acquired 100–1500 m/z for 25 ms in ‘high sensitivity’ mode. The following switch criteria were used: charge: 2+ to 4+; minimum intensity: 250 counts per second (cps). Up to 100 ions were selected for fragmentation after each survey scan. Dynamic exclusion was set to 15°s. The system sensitivity was controlled by analysing 0.5 µg of K562 trypsin digestion (Sciex), in these conditions, 2319 proteins were identified (FDR < 1%) in a 45-min gradient.

### Database search

ProteinPilotv5.0. search engine (ABSciex) default parameters were used to generate peak lists directly from 6600 plus TripleTOF wiff files. The Paragon algorithm [19] of ProteinPilot v 5.0 was used to search the SwissProt-200602 DB (562246 sequences) with the following parameters: trypsin specificity, IAM cys-alkylation, taxonomy not restricted, and the search effort set to rapid without FDR analysis. A ProteinPilot unused score above 1.3 was considered significant, which is the equivalent to a protein confidence threshold of over 95%. Only the proteins present after filtering the taxonomy to SARS-CoV-2 were analysed.

### Western blot analysis of VLPs and cell lysates

The quality and purity of the VLPs were assessed by SDS-PAGE. To detect the c-myc-tagged N protein, protein samples were transferred to nitrocellulose membranes (wet transfer 100 V (constant) 50 min at 4°C). Cellular debris contamination in the preparations was assessed by monitoring the presence of Histone 3. Western blots were performed using a mouse monoclonal anti c-myc (Sigma) and a mouse raised anti-Histone 3 (Sigma), followed by an anti-mouse secondary antibody conjugated to horseradish peroxidase (GE Healthcare). Visualisation was done using an Image Quant LAS4000mini (GE Healthcare).

### Transmission electron microscopy (TEM)

The samples were analysed on a Hitachi HT7800 Transmission Electron Microscope (TEM). To be able to observe the samples, 300MESh grids with carbon, which had been given glow discharge, were used. After making the deposition, the material was contrasted with phosphotungstic acid (negative staining).

## Results

### Synthesis and purification of SARS-CoV-2 VLPs

**Figure 1.**
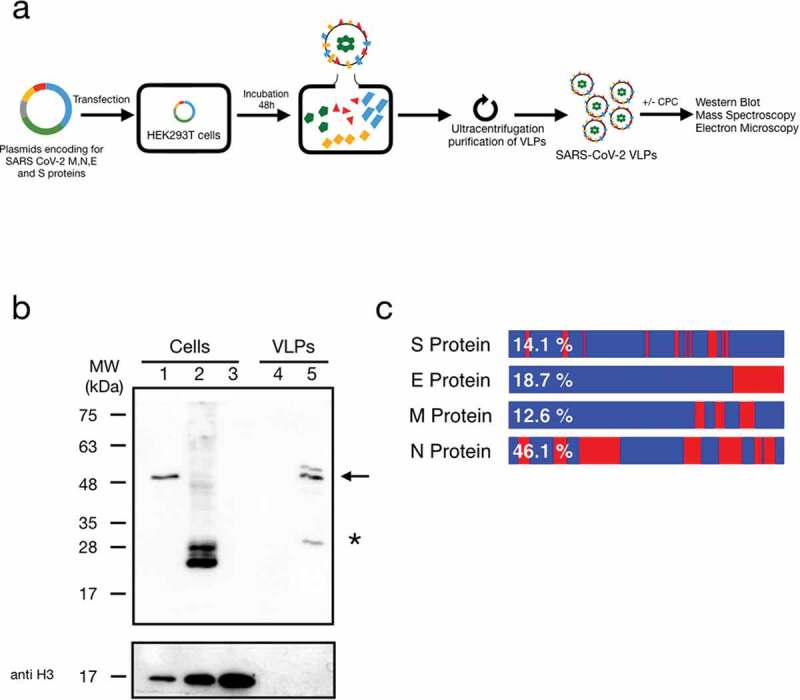
Expression of SARS-CoV-2 proteins in mammalian cells and VLPs. (a) Schematic representation of the workflow used for purification and analysis of SARS-CoV-2 VLPs. (b) Western blot identification of c-myc tagged N or M protein (upper panel, lanes 1 and 2). Arrow and asterisk indicate N and glycosylated M c-myc tagged proteins, respectively. Mock transfected cells were used as negative controls (lanes 3 and 4). Immunoblot analysis of the transfected cells and the purified VLPs showed the presence of the N protein only in the transfected cells (Figure 1(b), lanes 1, and 5) and not in the mock cells (Figure 1(b), lanes 3 and 4). To test for the presence of cell debris, all samples were blotted with an anti-Histone 3 antibody (bottom panel). (c) Schematic representation of the sequence regions and the protein percentage coverage by the LC-MS/MS for SARS-CoV-2 S, M, N, and E proteins. Red, protein regions covered; Blue, protein regions uncovered; percentages covered in each protein are shown in white.

SARS-CoV-2 VLPs were generated by transfecting HEK293T cells with plasmids encoding the nucleocapsid N (45.6 kDa), membrane M (25.1 kDa), envelope S (141.1 kDa) and E (8.3 kDa) proteins [17] (only N or M protein was c-myc tagged). At 48 h post-transfection culture media was collected and VLPs purified as previously described [18] (Figure 1(a)). Briefly, after cell incubation, supernatants containing VLPs were collected. Subsequently, VLPs were purified by ultracentrifugation. As expected, only cells transfected with c-myc tagged N or M proteins (Figure 1(b), lanes 1 and 2, respectively) were detected with c-myc antibody. The presence of N and M labelled proteins, in the individually purified but subsequently mixed fractions, were also confirmed (Figure 1(b), lane 5). Consequently, in the following experiments, the non-membrane N protein (with a c-myc label) was selected as a marker for the VLPs. To prove that cellular debris were not contained in our VLP preparation, we analysed the samples for the presence of Histone 3 (H3) (Figure 1(b), bottom panel). Extracts from transfected cells were included as positive controls. The absence of H3 signal in the VLPs (lanes 4 and 5) was a confirmation of the purity of the preparations.

Before protein identification, samples (VLP preparations) were deglycosylated using Peptide:N-glycosidase F (PNGase-F, an amidase that cleaves between the innermost GlcNAc and asparagine residues of high mannose, hybrid, and complex oligosaccharides from N-linked glycoproteins) treatment [20]. After the PNGase-F treatment samples were submitted for protein identification by LC-MS/MS. Peptides from all the SARS-CoV-2 transfected proteins (that is S, E, N, and M) were identified by LC-MS/MS in all four VLP preparations (Figure 1(c)). Supplementary Table S1 lists the sequence, molecular weight, and location on the sequence of the identified virus-derived peptides. The quality and percentage of coverage of the MS identifications are shown in Figure 1(c).

### Effect of CPC on the integrity of VLPs

**Figure 2.**
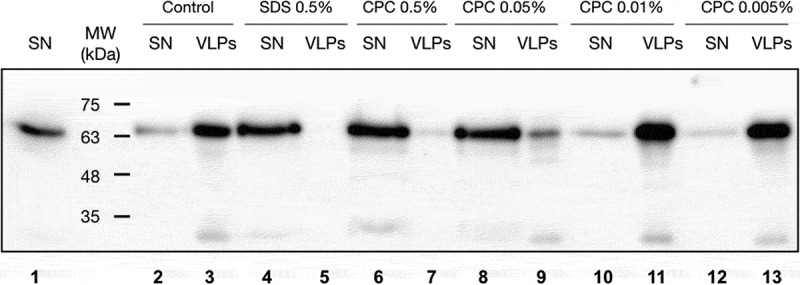
Western blot of SARS-CoV-2 VLPs treated in presence/absence of CPC. VLPs purified from the media of cells transfected with N-cmyc, M, S and E proteins were incubated with different concentrations of CPC; PBS buffer and SDS were used as controls. Then, the samples were ultracentrifuged, and two fractions were recovered, a soluble (supernatant, SN) one and a sedimented fraction (VLPs fraction). The samples were analysed by SDS-PAGE, and the presence of the tagged N-protein molecules were revealed by anti c-myc antibody immunoblotting.

Next, we examined the effect of CPC (cetylpyridinium chloride) detergent on the integrity of SARS-CoV-2 VLPs. VLPs recovered from purification process were incubated with CPC (from 0.5% to 0.0005%) or SDS (0.5%) or TBS for 5 min. Then, samples were ultracentrifuged, and the supernatant and pellet were analysed. As before, SARS-CoV-2 proteins were extracted and the c-myc tagged N-protein was analysed by Western blot, using a c-myc antibody (Figure 2). An increase of the viral protein content in the supernatant fraction was detected in the presence of a higher CPC concentration (Figure 2, lanes 6–8), indicating that at concentrations ≥0.05% the VLPs are disrupted by the detergent, facilitating content release into the supernatant. Similar results were observed when samples were treated with SDS (Figure 2, lanes 4 and 5), indicating once more that VLPs are disrupted under these conditions. At CPC concentrations of below 0.05% (Figure 2, lanes 10–14) the integrity of the viral particles was not affected. In these conditions, viral proteins were found in the pellet after CPC treatment and centrifugation (lanes 11 and 13). Similar results were obtained after mock treating of the VLPs (Figure 2, lanes 2 and 3). Together, this result demonstrates that CPC is active and induces a SARS-CoV-2 membrane disaggregation at concentrations above its cmc (critical micelle concentration, 0.042% in water media) [21].

**Figure 3.**
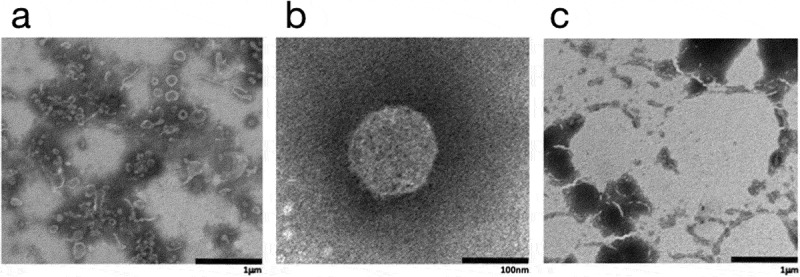
Morphological evaluation of SARS-CoV-2 VLPs. (a) TEM images of SARS-CoV-2 VLPs 48 h after co-transfection of S, M, E, and N protein containing plasmids. (b) Magnified VLP recovered in non-treated conditions. (c) TEM images of SARS-CoV-2 VLPs after CPC incubation.

Next, we evaluated the effect of CPC on the SARS-CoV-2 VLPs by means of transmission electron microscopy (TEM). Briefly, supernatants from incubated cells were collected by ultracentrifugation and incubated with/without CPC (0.05% in PBS). In the absence of CPC, SARS-CoV-2 VLPs appeared to be spherical (with a small percentage of filamentous particles) (Figure 3(a,b)). The average diameter of SARS-CoV-2 VLPs were around 223 ± 73 nm (Figure 3(a,b)). In contrast, SARS-CoV-2 VLPs treated with CPC (0.05%), lost their structural integrity, and thus were not detected by TEM (Figure 3(c)), suggesting that CPC is promoting membrane disaggregation.

## Discussion

As SARS-CoV-2 continues to spread, we must continue to build our fundamental understanding of its molecular virology. In this work, we utilised the ability of viral structural proteins to produce VLPs and found that CPC was sufficient to disaggregate VLPs in a concentration-dependent manner. Additionally, this highlights the need for further examination of the interaction between CPC and pathogenic viruses commonly present in the oral cavity.

Previously, transient co-expression of the four SARS-CoV-2 structural proteins in mammalian cell cultures have been shown to produce self-assembling VLPs which can be collected, purified, and used to study the molecular biology of the virus [17]. Multiple aspects of the viral life cycle of SARS-CoV-2 such as assembly, budding, egress, and entry have been studied using VLPs [3]. While these VLPs were both morphologically and functionally similar to SARS-CoV-2 virions, they do not contain the viral genome, are non-infectious, and thus can be used in a BSL-2 setting. In the present study, we generated VLPs by expressing the four SARS-CoV-2 structural proteins in mammalian cells (HEK293T). Then, we tested the integrity of SARS-CoV-2 VLPs in the presence of CPC, at previously known inhibitory concentrations [8]. The disaggregation of SARS-CoV-2 VLPs observed by immunoblotting was confirmed by electron microscopy. The present data suggest that CPC is a potential anti-SARS-CoV-2 inhibitor. The more plausible mechanism for the observed antiviral activity of mouth rinses and their active ingredients is the promotion of membrane disaggregation, although other potential mechanisms to inhibit capsid virus assembly have been the subject of speculation [22].

The results suggest that some types of toothpaste and mouthwash containing CPC could help to reduce the spread of SARS-CoV-2, by temporarily decreasing the number of competent virions in the mouth. Although SARS-CoV-2 transmission can occur by activities involving the oral cavity, such as speaking, breathing, coughing, sneezing and even singing [23,24], most attention has been focused on the nasal–lung axis of infection [25]. Oral manifestations, such as taste loss, dry mouth and oral lesions, are evident in about half of COVID-19 cases [26–28]. This is critical because, if these are sites of early infection, they could play an important role in transmitting the virus to the lungs or the gastrointestinal tract via saliva, as has been suggested for other microbial-associated diseases, such as pneumonia [29] and inflammatory bowel diseases [30,31]. Reducing the so-called ‘viral load’ could have important clinical consequences, particularly in dentistry, hence, multiple medical organisations and dental professionals have launched protocols and clinical recommendations to include this measure. Among the latter, the use of cetylpyridinium chloride (CPC) stands out as a prophylactic measure to reduce viral load in the oral cavity prior to dental procedures, with the aim of preventing the transmission of viral diseases in the dental clinic [32]. In addition, offering CPC mouthwashes could be an easy front-line strategy against the pandemic since vaccination rates in under-developed countries are still extremely low [33]. Moreover, given the high effectiveness of rinsing for 30–60 seconds with mouthwash, we underscore the importance of this approach. We hypothesise that CPC, a main active compound in mouth rinses may block infection by altering/disrupting viral envelopes (membranes).

CPC is a key ingredient of many commercial types of mouthwashes, which functions as an antiseptic that kills bacteria and other organisms, such as viruses [34]. One of the key features of CPC surfactant that enables it to target a diverse group of enveloped viruses (like Hepatitis B [22]) is its unique structure containing a hydrophobic hexadecyl membrane anchor group that can perturb lipid bilayers. Presumably, these effects are due to the incorporation of surfactant monomers into the viral lipid envelope inducing a disorder and damage of the lipid bilayer [35]. Human influenza (H3N2) and avian influenza virus strains (H5N3) were investigated with respect to the effect of the surfactants potassium oleate (KOl), SDS, and sodium laureth sulphate (SLES) [36]. Another nice example is the solubilization of the Semliki Forest Virus (SFV) membrane upon addition of the anionic surfactant SDS, which was studied by Becker et al. by density gradient centrifugation [37]. Different authors had proposed the use of surfactant mixtures (Triton X-100 and CPC) to be considered as candidates for inactivation Ebola virus [38]. Finally, a study showed that the administration of artificial pulmonary surfactant to mice infected with the H1N1 influenza virus in combination with antibody treatment increased the survival rate substantially. This effect was attributed to the surfactant inhibiting alveolar collapse and diffuse alveolar damage in the lungs, thereby preserving lung function for oxygenation [39].

Here, we identified CPC as a novel SARS-CoV-2 active compound using *in vitro* systems and demonstrated that CPC induces the disassembly of SARS-CoV-2 virus-like particles. These effects were confirmed by electron microscopy. In addition, analogous experiments using CPC diluted in filtered saliva showed similar antiviral results [40]. Future experiments will discern if the addition of compounds like CPC to mouthwashes is a valid strategy to fight against other enveloped virus, such as other coronaviruses [7].

Further studies are needed to unravel the relationship between viral load in the oral cavity and the severity of COVID-19 symptoms. These studies will help to improve our current understanding of the relationship between the oral cavity’s susceptibility to SARS-CoV-2 infection and the stage and progression of COVID-19.

## Supplementary Material

Supplemental Material
