# Supplementary material for: Cetylpyridinium chloride promotes disaggregation of SARS-CoV-2 virus-like particles
Source: J Oral Microbiol. 2022 Jan 24;14(1):2030094. doi: 10.1080/20002297.2022.2030094 (PMC8788378; doi:10.1080/20002297.2022.2030094)
Supplement: Supplemental Material [file ZJOM_A_2030094_SM2594.docx]

Table Supplementary 1

| **S** **Protein (*Spike*):** 1273 residues | | |  |
| --- | --- | --- | --- |
| UniProt Code: P0DTC2 – SPIKE_SARS2 | | |  |
| Description: *Spike glycoprotein* | | |  |
| Sequence | MW | Sequence Position | |
| RFDNPVLPFNDGVYFASTEK | 2315,1223 | 78 – 97 | |
| SYLTPGDSSSGWTAGAAAYYVGYLQPR | 2837,3296 | 247 – 273 | |
| FASVYAWNR | 1112,5403 | 347 – 355 | |
| VYSTGSNVFQTR | 1357,6626 | 635 – 646 | |
| ALTGIAVEQDKNTQEVFAQVK | 2288,2012 | 766 – 786 | |
| SFIEDLLFNK | 1224,6390 | 816 – 825 | |
| QYGDCLGDIAAR | 1337,6034 | 836 – 847 | |
| LIANQFNSAIGK | 1274,6982 | 922 – 933 | |
| IQDSLSSTASALGK | 1376,7147 | 934 – 947 | |
| LQDVVNQNAQALNTLVK | 1867,0164 | 948 – 964 | |
| LDKVEAEVQIDR | 1413,7463 | 984 – 995 | |
| LQSLQTYVTQQLIR | 1689,9414 | 1001 – 1014 | |

| **E Protein** (*Envelope*): 75 residues | | |
| --- | --- | --- |
| Código UniProt: P0DTC4 – VEMP_SARS2 | | |
| Description: *Envelope small membrane protein* | | |
| Sequence | MW | Sequence Position |
| VKNLNSSRVPDLLV | 1552,8937 | 62 – 75 |

| **M Protein** (*Membrane*): 222 residues | | |  |
| --- | --- | --- | --- |
| Código UniProt: P0DTC5 - VME1_SARS2 | | |  |
| Description: *Membrane protein* | | |  |
| Sequence | MW | Sequence Position | |
| IAGHHLGR | 859,4777 | 151 - 158 |  |
| EITVATSR | 875,4713 | 167 - 174 |  |
| VAGDSGFAAYSR | 1199,5571 | 187 - 198 |  |

| **N Protein** (*Nucleoprotein*): 419 residues | | |  |
| --- | --- | --- | --- |
| Código UniProt: P0DTC9 - NCAP_SARS2 | | |  |
| Description: *Nucleoprotein* | | |  |
| Sequence | MW | Sequence Position | |
| ITFGGPSDSTGSNQNGER | 1823,7922 | 15 - 32 | |
| GQGVPINTNSSPDDQIGYYRR | 2336,1145 | 69 - 89 | |
| WYFYYLGTGPEAGLPYGANK | 2266,0735 | 108 - 127 | |
| DGIIWVATEGALNTPK | 1683,8832 | 128 - 143 | |
| DHIGTRNPANNAAIVLQLPQGTTLPK | 2738,4827 | 144 - 169 | |
| NPANNAAIVLQLPQGTTLPK | 2059,1426 | 150 - 169 | |
| AYNVTQAFGR | 1125,5568 | 267 - 276 | |
| RGPEQTQGNFGDQELIR | 1943,9449 | 277 - 293 | |
| IGMEVTPSGTWLTYTGAIK | 2024,0288 | 320 - 338 | |
| LDDKDPNFKDQVILLNK | 2014,0735 | 339 - 355 | |
| KKADETQALPQR | 1383,7471 | 374 - 385 | |
| ADETQALPQR | 1127,5571 | 376 - 385 | |
| QQTVTLLPAADLDDFSK | 1860,9469 | 389 - 405 | |
